# Supplementary material for: Carcinoembryonic Antigen Expression in Human Tumors: A Tissue Microarray Study on 13,725 Tumors
Source: Cancers (Basel). 2024 Dec 3;16(23):4052. doi: 10.3390/cancers16234052 (PMC11640007; doi:10.3390/cancers16234052)
Supplement: Supplementary file 1 [file cancers-16-04052-s001.zip › Supplementary Table S1.pdf]

Organ system	Cell type	CEA staining
**Respiratory system**	Respiratory epithelium	Variable staining of epthelial cells, primarly in goblet cell.
	Lung	No staining.
**Gastrointestinal Tract**	Esophagus	Moderate to strong staining of squamous epthelial cells.
	Salivary glands	Weak to moderate staining, primarly in in mucimous cells
	Stomach	Moderate to strong staining in the surface cells and the upper half of crypts.
	Carcinoma of the anal canal	Strong staining in transitional epithelium
	Duodenum, mucosa	Moderate staining of epthelial cells.
	Duodenum, brunner gland	No staining.
	Colon descendes, mucosa	Strong staining is seen in epithelial cells of the colon mucosa. The intensity is highest in the surface epithelium.
	Rectum	Strong staining in epithelial cells of the rectum mucosa. The intensity is highest in the surface epithelium.
	Small intestine	Moderate staining of epthelial cells.
	Liver	No staining.
	Gallbladder	No staining.
	Pancreas	No staining.
	Kidney	No staining.
**Genitourinary tract**	Urothelium	No staining.
	Prostate	No staining.
**Male genital tract**	Seminal vesicles	No staining.
	Testis	No staining.
	Epididymis	Strong staining of epthelial cells.
	Breast	No staining.
**Female genital tract**	Uterus, ectocervix	Moderate to strong staining occurs in the superficial cell layers of the squamous epithelium of the ectocervix.
	Uterus endocervix	No staining.
	Uterus, endometrium	No staining.
	Fallopian Tube	No staining.
	Ovary	No staining.
	Placenta	No staining.
	Amnion	No staining.
	Chorion	No staining.
	Skin	No staining.
**Skin**	Eccerine glands	Staining of luminal membrane.
	Heart	No staining.
**Muscle and connective tissues**	Skeletal	No staining.
	Fat	No staining.
	Bone marrow	No staining.
**Lymphoid tissue**	Lymph node	No staining.
	Spleen	No staining.
	Thymus	Strong staining in corpuscles of Hassall's.
	Tonsil	Strong staining squamous epithelial cells in tonsil.
	Cerebrum	No staining.
**Brain**	Cerebellum	No staining.
	Thyroid	No staining.
**Endocrine Tissues**	Parathyroid	No staining.
	Adrenal gland	No staining.
	Pituitary gland	No staining.
