# Supplementary material for: Carcinoembryonic Antigen Expression in Human Tumors: A Tissue Microarray Study on 13,725 Tumors
Source: Cancers (Basel). 2024 Dec 3;16(23):4052. doi: 10.3390/cancers16234052 (PMC11640007; doi:10.3390/cancers16234052)
Supplement: Supplementary file 1 [file cancers-16-04052-s001.zip › Supplementary Table S2.pdf]

	HPV status	CEA i	
		n	negative (%)
All squamous cell cancers	negative	285	64.2
	positive	239	51.9
Oral squamous cell carcinoma	negative	65	67.7
	positive	13	84.6
Squamous cell carcinoma of the pharynx	negative	21	66.7
	positive	34	55.9
Squamous cell carcinoma of the larynx	negative	50	52.0
	positive	9	22.2
Squamous cell carcinoma of the cervix	negative	10	50.0
	positive	68	30.9
Squamous cell carcinoma of the vagina	negative	15	60.0
	positive	15	60.0
Squamous cell carcinoma of the vulva	negative	51	64.7
	positive	26	65.4
Squamous cell carcinoma of the penis	negative	30	66.7
	positive	44	68.2
Squamous cell carcinoma of the skin	negative	38	76.3
	positive	1	100.0
Squamous cell carcinoma of the anal canal	negative	5	60.0
	positive	29	48.3

mmunostaining result			
weak (%)	moderate (%)	strong (%)	P
20.4	4.6	10.9	0.0281
24.3	7.5	16.3	
15.4	9.2	7.7	0.2186
15.4	0.0	0.0	
19.0	0.0	14.3	0.3449
23.5	8.8	11.8	
22.0	4.0	22.0	0.1926
33.3	22.2	22.2	
30.0	0.0	20.0	0.2121
20.6	14.7	33.8	
13.3	6.7	20.0	0.2884
33.3	0.0	6.7	
25.5	2.0	7.8	0.2028
34.6	0.0	0.0	
20.0	6.7	6.7	0.9846
20.5	6.8	4.5	
21.1	0.0	2.6	0.7662
0.0	0.0	0.0	
20.0	20.0	0.0	0.0991
27.6	0.0	24.1	
