# Supplementary figures and images for: Carcinoembryonic Antigen Expression in Human Tumors: A Tissue Microarray Study on 13,725 Tumors
Source: Cancers (Basel). 2024 Dec 3;16(23):4052. doi: 10.3390/cancers16234052 (PMC11640007; doi:10.3390/cancers16234052)

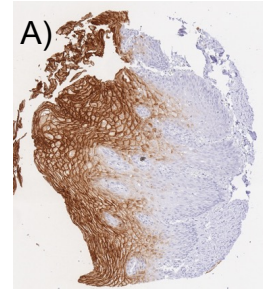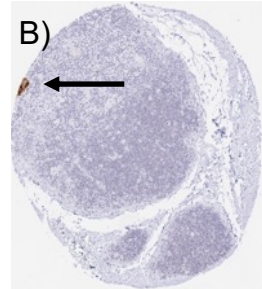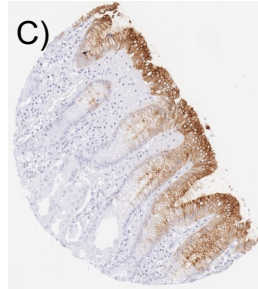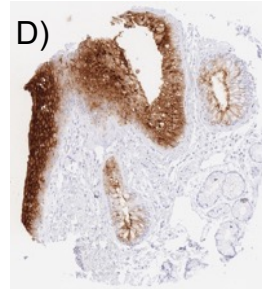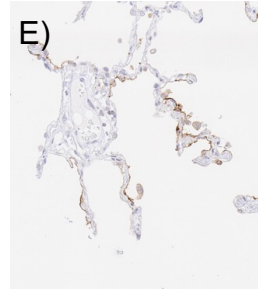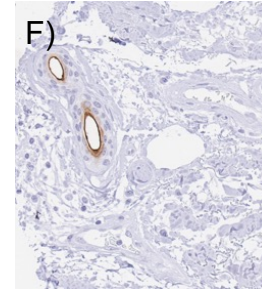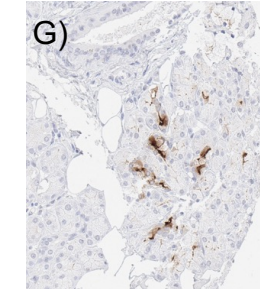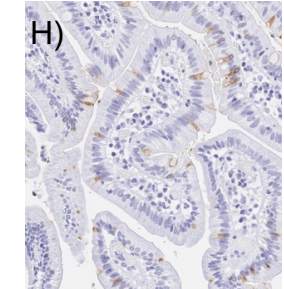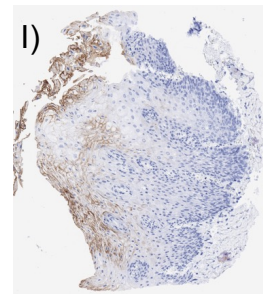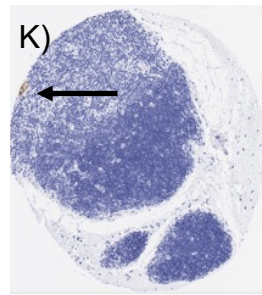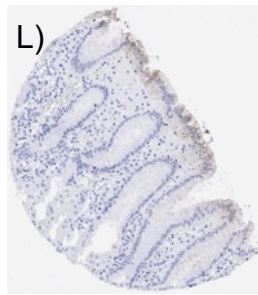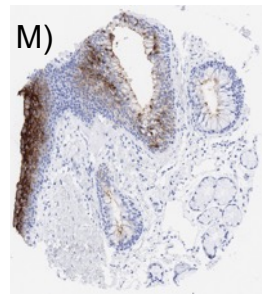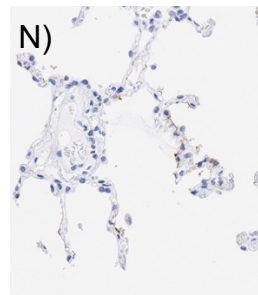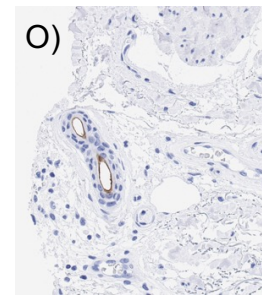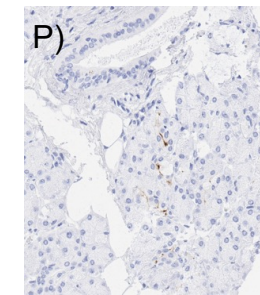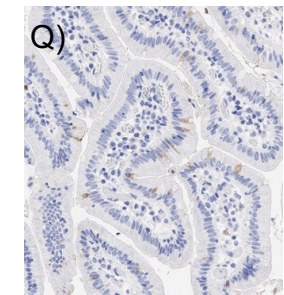

Supplement: Supplementary file 1 [file cancers-16-04052-s001.zip › Suppl Figure S1_CEA.pdf]
